# A Novel Heavy Domain Antibody Library with Functionally Optimized Complementarity Determining Regions

**DOI:** 10.1371/journal.pone.0076834

**Published:** 2013-10-08

**Authors:** Ole Aalund Mandrup, Niels Anton Friis, Simon Lykkemark, Jesper Just, Peter Kristensen

**Affiliations:** 1 Department of Engineering, Aarhus University, Aarhus, Denmark; 2 Department of Molecular Biology and Genetics, Aarhus University, Aarhus, Denmark; 3 Department of Clinical Medicine, Aarhus University, Aarhus, Denmark; 4 Sino-Danish Centre for Education and Research, Aarhus, Denmark; Centro Nacional de Biotecnologia - CSIC, Spain

## Abstract

Today a number of synthetic antibody libraries of different formats have been created and used for the selection of a large number of recombinant antibodies. One of the determining factors for successful isolation of recombinant antibodies from libraries lies in the quality of the libraries i.e. the number of correctly folded, functional antibodies contained in the library. Here, we describe the construction of a novel, high quality, synthetic single domain antibody library dubbed Predator. The library is based on the HEL4 domain antibody with the addition of recently reported mutations concerning the amino acid composition at positions critical for the folding characteristics and aggregation propensities of domain antibodies. As a unique feature, the CDR3 of the library was designed to mimic the natural human immune response by designating amino acids known to be prevalent in functional antibodies to the diversity in CDR3. CDR randomizations were performed using trinucleotide synthesis to avoid the presence of stop codons. Furthermore a novel cycle free elongation method was used for the conversion of the synthesized single stranded DNA containing the randomized CDRs into double stranded DNA of the library. In addition a modular approach has been adopted for the scaffold in which each CDR region is flanked by unique restrictions sites, allowing easy affinity maturation of selected clones by CDR shuffling. To validate the quality of the library, one round phage display selections were performed on purified antigens and highly complex antigen mixtures such as cultured eukaryotic cells resulting in several specific binders. The further characterization of some of the selected clones, however, indicates a reduction in thermodynamic stability caused by the inclusion the additional mutations to the HEL4 scaffold.

## Introduction

**Figure 1:**
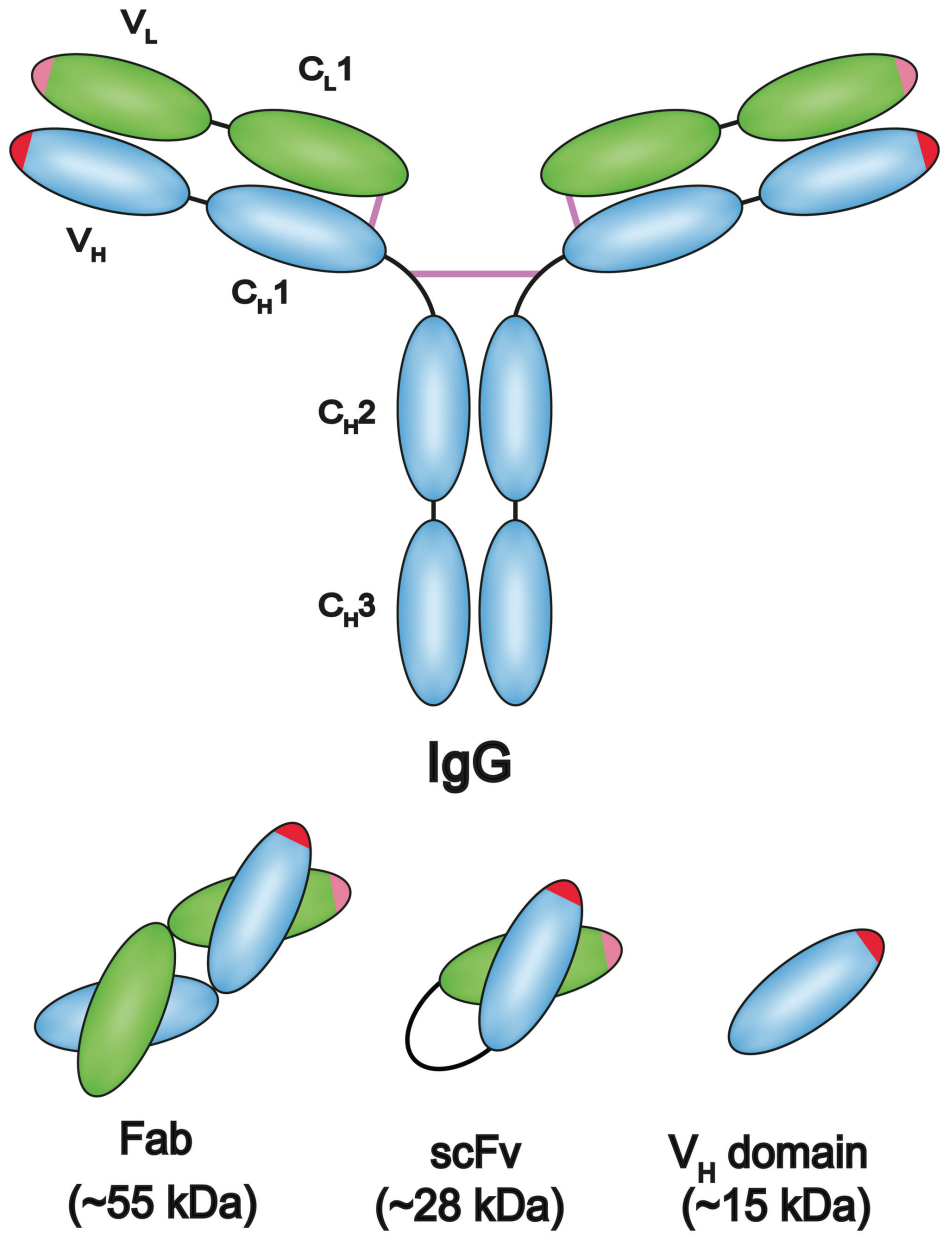
Common recombinant antibody formats. Different fragments from the immunoglobulin are often used in recombinant formats in libraries, especially the Fab fragment, the scFv and the variable domain of the heavy chain have been used repeatedly.

Recombinant antibody technology relies on the manipulation of genes encoding antibodies outside of the human body. The choice of recombinant antibody scaffold most often lies between the fragment antigen binding (Fab), the single-chain variable fragment (scFv), or the domain antibody (dAb) scaffold, all of which have been shown to be suitable for the phage display technique (Figure 1) [1-3]. The different scaffolds have their strengths and weaknesses, and especially stability and ease of production in *E. coli* differs between the scaffolds. Most often the variable part of the antibody has contributions from both the heavy and the light polypeptide chain, thus giving rise to Fab or scFv scaffolds. The dAbs, which are derived from antibodies having only the heavy chain, have been found to be naturally occurring in camelids and sharks. They can also be observed in humans in connection with certain myelomas [4]. The use of fully human domain scaffolds has been hampered by the strong tendency of the variable gene repertoires to form aggregates. Human heavy chain variable fragments (Vh) have therefor previously been compared to those found in camelids [5]. Mutational studies of the human Vh have also strongly aided to the understanding of the factors leading to these problems, and thereby to possible solutions [6-9]. Recently a human domain antibody library using the HEL4 scaffold was counter-selected for aggregation and the CDR regions of the resulting clones were then sub-cloned and used for generating a new library with diversity in all three CDR regions. This library is now available from Source Bioscience, UK [10]. Further studies of domain antibodies have underscored that the regions governing the aggregation propensity are located in or adjacent to the CDR regions. This was shown more detailed in sequence analysis of unselected clones and clones counter selected for aggregation. The preferences for certain amino acids especially in the CDR1 region after the selection indicated that the CDR1 region was central with regard to the aggregation characteristics [6,11]. Investigation of the HEL4 antibody has revealed that especially the “DED” amino acid triad at position 31 to 33 of the CDR1 (Kabat numbering) is central for the folding-characteristics of HEL4. Previously, it has also been indicated that the adjacent “I” in position 29 can be replaced by an aspartic acid thus improving the folding properties. The CDR2 and CDR3 did, however, not seem to contribute significantly to the aggregation resistance properties of HEL4 in this study [11].

The determinants of antibody binding specificity are predominantly located in the complementarity determining regions (CDRs). Consequently, these are the areas in which the antibody library diversity is focused. Different categories of libraries can be defined ranging from immunized over naïve and semisynthetic to fully synthetic [12]. An immunized library relies on the natural antibody affinity maturation of the host and therefore an immunized library would be expected to contain multiple high affinity binders against the antigen used in the immunization. Naïve libraries are based on genetic material obtained from the host before being exposed to any immunogenic substances, or from a host which appear healthy without a compromised immune system, therefore a naïve library is not supposed to contain bias for binders to any particular antigen [13-15]. . That the naïve library has not been through the natural antibody maturation in the host should in theory greatly improve the chances of isolating a wider variety of binders and anti-self antibodies directed against the host’s own naturally occurring molecules [16]. By introducing diversity artificially it is possible to circumvent any bias introduced by the host immune system. It also allows for specific introduction of diversity at defined positions, e.g. positions known to be important in antibody-antigen interactions [17,18]. In the case of synthetic generation of diversity using trinucleotide synthesis, the method allows for the full control of the amino acid composition in the CDRs. This has, for example, been used to generate minimalistic antibody libraries constructed with as few as two amino acids in the randomized CDR region [19,20]. Semi-synthetic libraries with different combinations of synthetically and naturally host derived diversity have also been created.

Unlike the immunized and naïve libraries, the synthetic and semisynthetic libraries can be made using only one or few of the antibody framework repertoires. This reduces the contribution to diversity from the interface residues bordering the CDRs. It has, however, been shown, both in vitro and in vivo, that some variable (V) segments are much more commonly used in functional antibodies than others. Furthermore there are great differences in the stability and suitability for bacterial expression between different frameworks [21,22].

Following the creation of large libraries of recombinant antibodies, systems, which allow sorting of the libraries, need to be introduced and here display of the recombinant antibodies on the surface of the filamentous bacteriophage (phage) have played a pioneering role. The application known as “phage display” was first developed by G.P Smith in 1985 [23]. In phage display a foreign protein is expressed in fusion with one of the phage capsid proteins by cloning the foreign DNA sequence into the same reading frame as the gene of the chosen capsid protein. The major strength of phage display is the physical coupling between the phenotype (the fusion protein) and the genotype (the DNA inside the virion encoding the fusion protein). This coupling allows for high throughput selection of desirable properties of the fusion protein from libraries with billions of variants. There are two very important aspects of phage display selection of recombinant antibodies, namely the nature and quality of the material containing the antigen selected against, and perhaps more overlooked the quality and design of the displayed antibody library itself. Numerous phage display antibody libraries have been created over the years with many different specialized features, and the creation of new libraries continues as knowledge and technology expands [10,15,18,20,24-26]. In this paper we report the construction of a novel domain antibody library with restricted randomizations at 4 and 7 positions in the CDR2 and CDR3, respectively. This was done by the use of trinucleotide synthesized oligonucleotides and an innovative PCR free annealing strategy designed to avoid clonal bias due to sequence and amplification of non-unique clones caused by the PCR cycling. The diversity of the library was designed to reflect the amino acid composition of CDR regions from known functional human antibody clones, combined with additional knowledge of binding characteristics from specific amino acids. The library was furthermore designed to be highly modular by including unique restriction sites surrounding all three CDRs, the leader peptide, and the tag region. The library was based on the aggregation resistant HEL4 domain antibody scaffold with the inclusion of a hydrophilic mutation at position 29 previously reported to improve solubility.

## Materials and Methods

### Predator library construction

**Figure 2:**
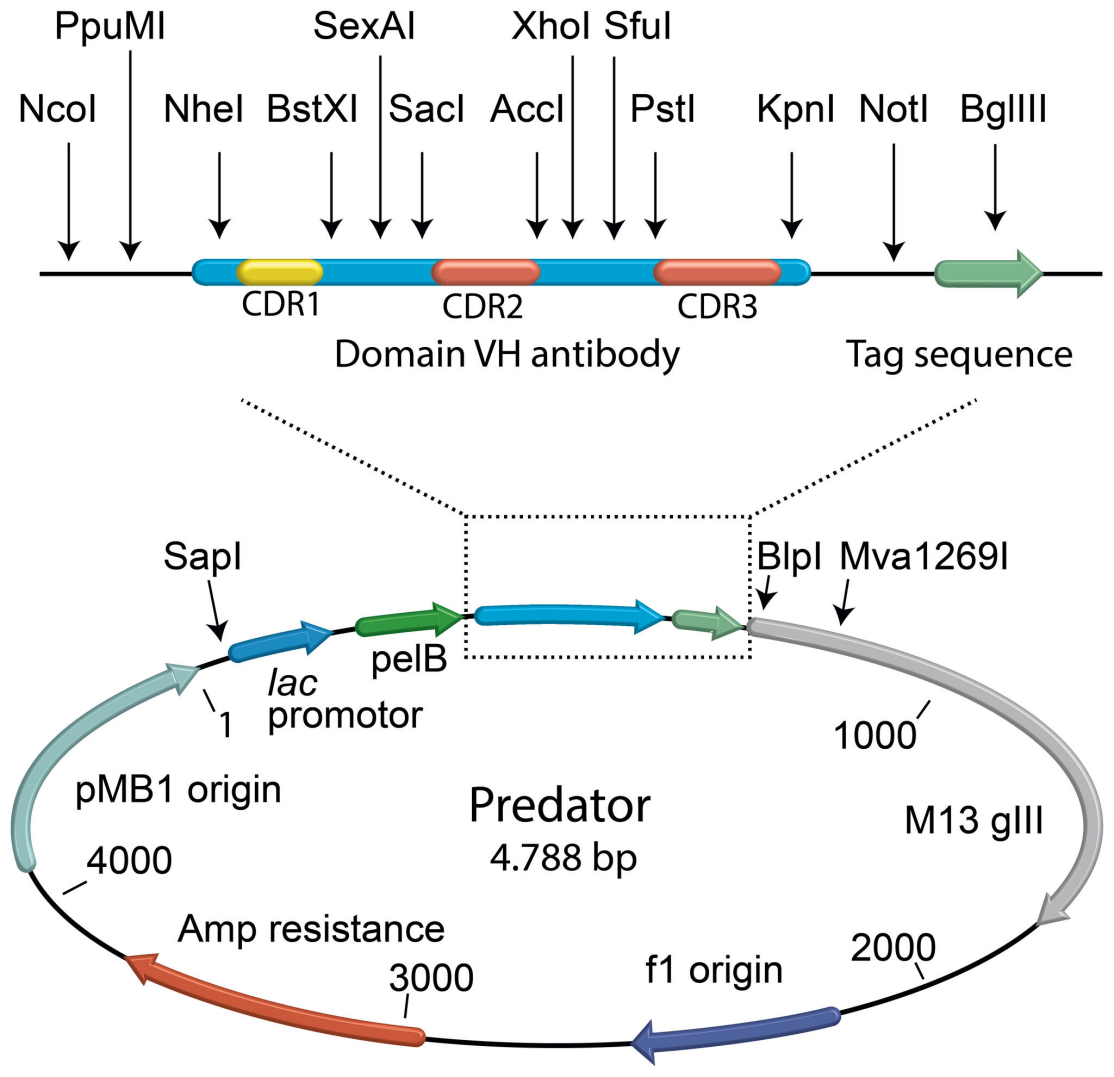
Vector map of the Predator library. The Predator vector map with the most important components marked. At the top of the figure an enlargement of the antibody region with the unique restriction sites and cassettes surrounding the CDR loops is shown.

All enzymes used were purchased from Fermentas (Sankt Leon-Rot, Germany) unless otherwise specified. The Predator library was constructed in the pHEN1 vector by insertion of a synthesized dAb gene between the unique SapI and Mva1269I restriction sites. This means that the library includes ampicillin resistance gene, the f1 phage origin of replication, most of the phage gene III and the bacterial origin of replication from the original pHen1 vector [3]. The dAb scaffold gene was based on the HEL4 sequence (GenBank: CQ761108.1) with silent mutations introduced to create unique restriction sites around the CDR regions. The dAb gene along with the flanking sequences were synthesized at Mr. Gene (Regensburg, Germany) according to our design (Figure 2). The synthesized dAb gene was digested with SapI and Mva1269I and ligated into pHEN1 to create the preliminary Predator vector. Finally an isoleucine in the framework region just prior to CDR1 was changed to an aspartic acid by site directed mutagenesis PCR.

**Figure 3:**
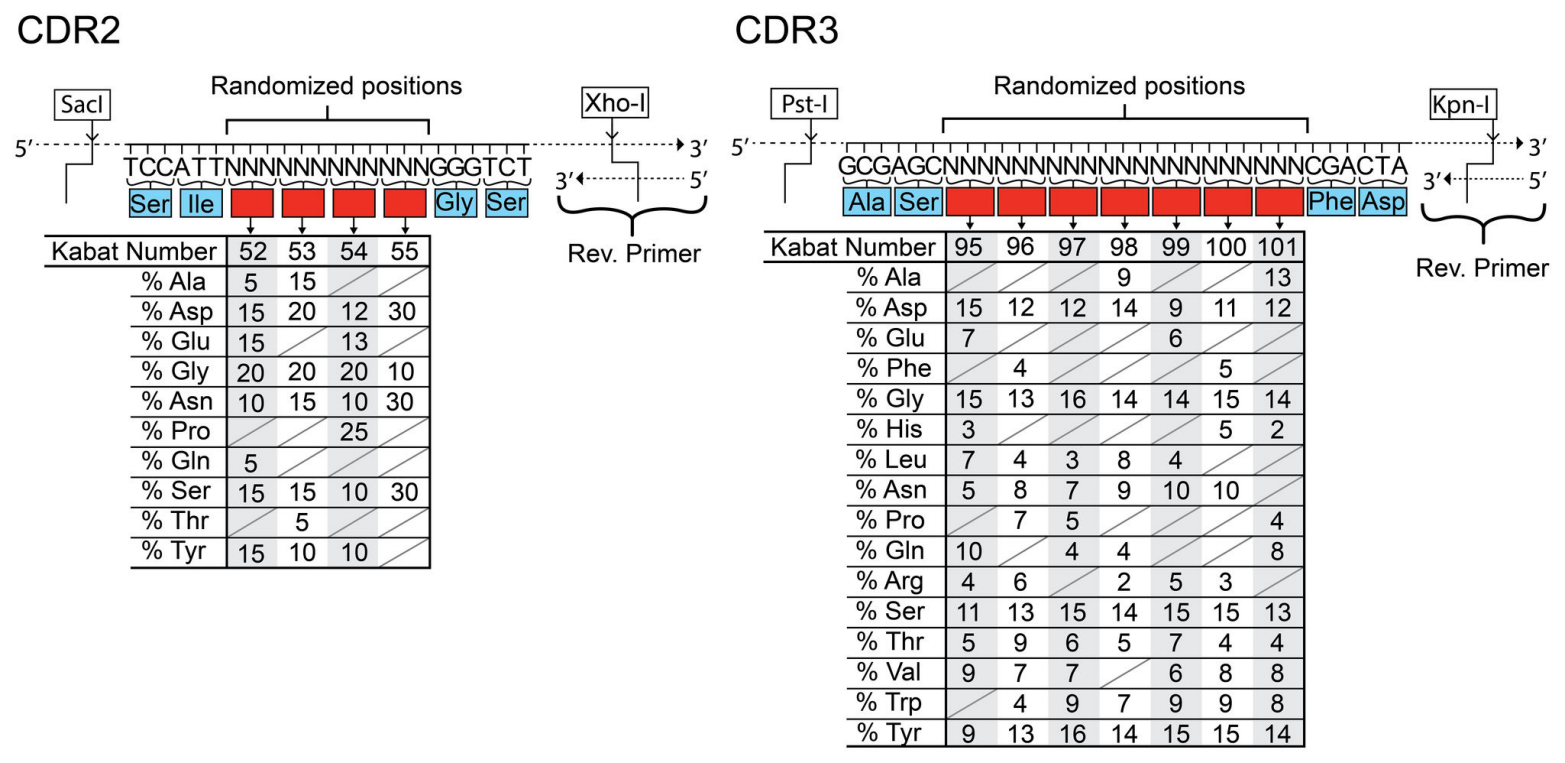
The diversity of the Predator library. The CDR2 and CDR3 of the library have been designed with specific frequencies designated to every amino acid used for all the randomized position (red boxes) in both CDR2 and CDR3 according to the different properties of the amino acids. The frequency of a given amino acid at a given position can be read from the table. The unique restriction sites used for cloning the CDR regions into the vector are shown flanking the sequences.

To generate the diversity of the library, inserts covering CDR2 and CDR3 were synthesized as single stranded DNA (ss-DNA) at ELLA Biotech (Martinsried, Germany) using trinucleotide “trimers” at the randomized positions. The CDR2 and CDR3 ss-DNA were prepared by annealing matching revers primers to their respective 3’ ends. Reverse primers were designed to have a melting temperature of approximately 68°C. The annealings were made in 1x PFU buffer by mixing the synthesized ss-DNA and matching reverse primers in a 1:2 molar ratio in PCR tubes. The mixtures were put in a 95°C water bath and allowed to cool slowly to room temperature. Following the annealing step PFU polymerase was added and the mixtures were incubated two hours at either 62°C for CDR2 or 58°C for CDR3 for elongation to occur. The elongated double stranded DNA (ds-DNA) inserts were then phenol extracted and alcohol precipitated according to standard protocol [27]. The CDR2 insert was double digested with SacI and XhoI restriction enzymes and the CDR3 insert was double digested with PstI and KpnI (Fig. 3). Following digestions both inserts were purified after separation in a 2% agarose gel [27]. The double stranded nature of the inserts was verified by digestion of a small sample with Mung bean nuclease (New England Biolabs, USA) and visualization on an agarose gel.

The Predator vector was prepared for receiving the CDR2 insert by double digestion with SacI and XhoI followed by dephosphorylation using shrimp alkaline phosphatase. The digested vector was then gel purified on a 1% agarose gel followed by electroelution, phenol extraction and alcohol precipitation. The purified vector was mixed in PCR tubes with CDR2 insert in a 1:3 molar ratio and ligated for 90 minutes at 22°C then 2 hours at 17°C followed by 2 hours at 8°C, producing the Predator CDR2+ repertoire. The ligation was phenol extracted, alcohol precipitated and dissolved in pure deionized water to a final concentration of 1µg/µl. 20µg of purified recombinant DNA, was electroporated into 350µl freshly prepared electro competent Novablue Gigasingles: (r_K12_^–^ m_K12_^+^) *thi-1 recA1 gyrA96* F'[*proA* ^*+*^ *B* ^*+*^ *acI* ^*q*^ *Z*Δ*M15*::Tc^R^)] (Merck KGaA, Germany). The transformed cells were plated on a large square 245x245 mm bioassay dishes (Nunc, Denmark) containing TYE, ampicillin and 2% glucose. Dilutions were made from the electroporation and likewise plated in order to titer the electroporation efficiency. Colonies were scraped off the agar plates and grown overnight in 2xTY bacterial growth media. Large scale plasmid DNA preparation was made of the Predator CDR2+ repertoire. Predator CDR2+ was double digested with PstI and KpnI and prepared for ligation as described previously. The purified vector was mixed in PCR tubes with CDR3 insert in a 1:10 molar ratio and ligated for 18 hours at 18°C in a PCR machine. The ligation was phenol extracted, alcohol precipitated and the pellet was dissolved in pure deionized water to a final concentration of 709ng/µl. For the electroporation, 21 µg of DNA was electroporated into 300µl freshly prepared Novablue Gigasingles. Five electroporations were performed and plated on a total of 10 large bioassay dishes containing TYE, ampicillin and 3% glucose. Dilutions were made from each of the five electroporations and likewise plated in order to titer the electroporation efficiency and determine the library size. The colonies were scraped off the plates and phages were produced as described elsewhere [28]. The remaining bacteria were mixed with glycerol until a final concentration of 10% and -80°C stocks were made of the library.

### Phage production

Phage particles were produced by superinfection with the KM13 helper phage, which contains a trypsin recognition site between domain D2 and D3 of the phage protein III [29]

Phages were either titrated by counting colony forming units (CFU) or phage titers were estimated by measuring phage absorbance at 269 nm and 320 nm followed by calculating the phage concentration as described by Barbas et al. [30].

### Estimating display level

Phage particles from the Predator library packaged with the KM13 helper phage were titrated by counting CFU from dilution series. In parallel the dilutions were incubated 30 min at 37°C with 1mg/ml trypsin prior to infection of TG1: *E. coli* K12 strain Δ(*lac-proAB*) *supE thi hsdD5*/F’ *traD36 proA+BlacIq lacZΔM15* (Source Bioscience, UK), in order to remove infectivity of phage particles not displaying antibody. Display level was then estimated based on the difference in CFU between trypsin treated and untreated phages.

### Single round selection on purified protein

For the selection on purified protein the antigen Hen Egg Lysozyme (Sigma-Aldrich, USA) was immobilized in a 4 ml MaxiSorp immunotube (Thermo, Fisher Scientific, USA). The incubation with the phage library used 2x 10^12^ phages in 3 ml of PBS with 4% w/v Marvel brand skimmed milk powder (4% MPBS) (Premier Foods, UK) and was carried out for 2 hours on a roller table at room temperature. The tube was washed with 75 ml of PBS with 0.05% Tween and then with 75 ml PBS. Phages were afterwards eluted with trypsin. The trypsinised phages were infected into 10 ml of exponentially growing TG1, and the infected cells were plated on TYE plates containing 1% w/v glucose and 100 µg/ml ampicillin. Individual clones were picked from the TYE plates into 96-well culture trays with 100 µl 2xTY with 1% glucose and 100 µg/ml ampicillin and grown over night at 37 °C in a shaking incubator.

### Eukaryotic cell handling and selections

For the selection on eukaryotic cells, Human Brain Vascular Pericytes (HBVP) (Sciencell Research Laboratories, USA) were grown in Pericyte medium (Sciencell, USA) at 37°C, 5% CO_2_ and 95% humidity. Cells of passage 4 were detached from a tissue culture flask by trypsination (Trypsin EDTA, Lonza, Denmark) and spun down at 800 g for 6 minutes. The cells were then resuspended in medium and 2x 10^5^ pericytes were seeded per well in a six well costar polystyrene cell culture plate (Corning incorporated, USA). The pericytes were grown 20-24 hours in pericyte medium in order to recover expression of surface antigens. The medium was aspirated and the cells were rinsed with PBS before being fixed in 2% paraformaldehyde (PFA) for 15 minutes at room temperature. Each well was incubated with 10^12^ phages in 2 ml of 2% MPBS and 10% Fetal Bovine Serum (Thermo Scientific, USA) for 2 hours on a shaking table at room temperature. Each well was then washed 18 times with 3 ml PBS before eluting bound phages with trypsin from Bovine pancreas (Sigma-Aldrich, USA). Eluted phages were used for infection into TG1 as described previously.

### ELISA screening with phage antibodies

Clones selected for binding to Hen Egg Lysozyme were screened in antibody-phage ELISA in 96-well MaxiSorp plats (Thermo, Fisher Scientific, USA) coated with either lysozyme or milk protein. Individual phage antibody clones were produced in 96 well culture plates and precipitated with poly ethylene glycol (PEG) 6000, 0.5 M NaCl. The precipitated phages were dissolved in 100 µl PBS per well and 50 µl of each phage solution was used in ELISA screening on immobilized lysozyme and milk protein. The phages were detected using anti-M13-HRP (GE Healthcare, Denmark), and TMB readymade substrate (Thermo, Fisher Scientific, USA). The chromogenic reaction was halted by addition of 50 µl of 1M H_2_SO_4_, and the absorbance was measured at 450 nm in a Biorad 550 microplate reader (Biorad, USA). The six clones giving the best ratios of signal on lysozyme over milk protein were tested further. Phages were produced from the six clones in 50 ml cultures, and purified by PEG precipitation. The binding to lysozyme and to milk protein was examined in dilution series ELISA.

Clones selected for binding to HBVP were likewise screened by antibody-phage ELISA. Approximately 10,000 HBVP per well were seeded in a 96-well cell culture plate (Corning, USA) and grown for 20-24 hours. Individual phage antibody clones were produced as described previously and tested against PFA fixed HBVP and wells coated with pericyte medium. Interesting clones were tested further by production of antibody-phage from 50 ml cultures. The binding to HBVP, human microvascular endothelial cells (HMEC1) (ATCC, USA), ASF-2 fibroblasts (adult skin fibroblast) [31], human vascular smooth muscle cells (primary explant culture from aorta) [32] and pericyte medium were evaluated in dilution series ELISA.

### Expression and purification of soluble antibodies

To express soluble antibody, the selected clones were sub-cloned into pET22b (Merck KGaA, Germany) using the NcoI and NotI restriction sites and transformed into BL21 Gold (Agilent Technologies, USA). Protein expression was induced by addition of IPTG at OD_600_ = 0.7-0.9 and cultures were incubated at 30°C for 14 hours in shaking incubator. The supernatant was prepared by sterile filtration (0.45µm) and the protein precipitated by addition of 30% w/v ammonium sulfate. Antibody was purified from precipitated protein on Protein A HP spintraps (GE Healthcare, Denmark) according to manufacturer’s protocol. Protein concentrations were measured using a Nanodrop 1000 spectrophotometer (Thermo, Fisher Scientific, USA). More than 95% purity was verified by SDS-PAGE.

### Antibody dilution series ELISA on lysozyme

The dAb lysozyme binders were diluted to a starting concentrations of 5 µM PBS with 3% BSA (Sigma-Aldrich, USA), and an 8 step 3-fold dilution series of 100 µl volumes was prepared. The assay was performed in NUNC maxisorb 96 well plates coated with lysozyme (50 µM in 100 µl PBS) and blocked with 3% BSA (Sigma-Aldrich, USA). Detection of bound antibody was done using HP conjugated Protein A (Invitrogen, USA) diluted 1:5000 and TMB ready made substrate, as described for the ELISA screening.

### Cell staining with soluble antibodies

HBVP or HMEC-1 were seeded in ibi, Treat µ-Slide VI 0.4 (Ibidi, Germany) and incubated overnight. Cells were then fixed in 2% PFA for 15 minutes and blocked with MPBS-Glycine solution (2% MPBS and 0.3 M Glycine). The cells were incubated with 30 µg of purified predator antibody per well for 2 hours at room temperature. Visualisation of the predator antibodies was accomplished by incubation with Protein A conjugated with Alexa Fluor 488 (Invitrogen, USA) and cell nuclei were stained by VECTASHIELD Mounting Medium with DAPI (Vector Labs, USA). Fluorescent images were obtained by a Leica DMI3000 B inverted microscope (Leica Microsystems, Germany).

### Circular dichroism (CD) spectroscopy of thermal unfolding

Melting temperature for the six lysozyme binders in dAb format was measured in a Jasco-810 CD spectrometer equipped with Peltier temperature controlled cuvette holder. The thermal unfolding was measured at protein concentrations of 18 µM in PBS, except clone B11 at 11.7 µM. Temperature ramping speed was set to 60°C/h. The thermal stability was monitored by the decay CD signal corresponding to secondary structure elements at 235 nm as described by Jespers et al. [33]. The Tm was determined from the curve inflection point by fitting the data to a sigmoidal curve equation using GraphPad Prism. When possible due to absence of irreversible aggregation upon heating, two heating curves were made for each antibody to determine the degree of refolding [34].

### Size exclusion chromatography

Protein A purified dAb C3 was run at 1ml/min in PBS over a C26 / 100 size exclusion column packed with Sephacryl S-100 mounted on an ÄKTA explorer FPLC unit in order to evaluate sample molecular size homogeneity. The protein was loaded and a chromatogram containing the evolution of the absorbance at 280 nm against the elution volume was recorded.

### Affinity measurement for the dAb C3

Isothermal titration calorimetry was performed on a Microcal iTC200 instrument (GE Healthcare, Denmark). Lysozyme and dAb C3 were dialysed against the exact same volume of PBS before use. The experiment was performed at 20°C with 350 µM antibody in the sample cell and adjusted to 4µl injections of 24.6 µM lysozyme. The resulting integrated injection peaks were fitted to a 1:1 binding isotherm and the resulting binding parameters (KD, stoichiometry, Active cell concentration) were calculated. The data representing an average of three independent measurements with corresponding standard deviation were calculated on the basis of these data. Figures were prepared using GraphPad Prism software.

## Results and Discussion

### Predator scaffold

The scaffold used for the Predator library is based on the V_H_3 3-23/DP47 germline segment of the HEL4 domain antibody, which has previously been shown to be resistant to aggregation [35]. As it was previously reported that mutation at position 29 from “I” to “D” can improve the solubility of a model V _H_3 domain antibody [11], this mutation was also included. Position 29 is known to have a strong preference for hydrophobic amino acids in V_H_3 [22]. There is, however, no evidence that a hydrophobic amino acid is required for the structural stability of antibodies [36]. As the CDR1 has been shown to be critical for the properties of the HEL4 antibody, randomizations were only made in CDR2 and CDR3. The scaffold was synthesized *de novo*, which allowed full control of the design with regard to the nucleotide sequence. In the design of the scaffold several unique silent restrictions sites were placed in the sequence, thereby making the scaffold highly modular. Restriction sites flanking the CDR2 and CDR3 were later used for cloning the oligonucleotide introducing diversity into the library (Figure 2). The restriction sites were preserved after the cloning of CDR2 and CDR3 so subsequent cloning would be possible e.g. for CDR walking or similar affinity maturation. Furthermore we designed the gene so that rare codons were avoided when this did not interfere with the placement of the unique restriction sites [37]. For the construction of the Predator library the pelB leader was used, as it has previously been shown that differences in efficiency between different leader sequences and export pathways used for phage display in *E. coli* appears to be minimal [38]. To increase display of fusion proteins and improve virion production a small stretch of nucleotides normally found in the sequence between the *lac* promoter and the pelB leader in the pHEN1 vector and several other commonly used phagemid vectors was removed [39]. In phage production from the modified phagemid we obtained a good display ratio of an estimated 6.4% (data not shown).

### The diversity

The diversities of the CDR2 and CDR3 sequences were designed to take into account important characteristics regarding the amino acids placed in the CDR regions. The composition of the CDR loops may have a significant influence on the antibody aggregation propensity. This is important to take into consideration when creating diversity for synthetic libraries as the natural selection in the immune cells is absent and the risk of aggregation of antibody clones might thus be increased. Propensities for aggregation were taken into account in the design of the CDR2 diversity, based on previously published work of the amino acid composition in CDR2 [6]. Generally the use of the most hydrophobic amino acids (i.e. V, I, L, M, and F) was kept at a moderate to low level (with a 0-25% likelihood of a hydrophobic amino acid at any given randomized position). This was done to lower the overall hydrophobicity of the CDRs thereby reducing the risk of aggregation [6], while at the same time allowing hydrophobic contributions to the antigen binding. Additional favorable contributions to binding, documented for tyrosine, serine and glycine, were taken into account in the design of both the CDR2 and CDR3 [40]. Positively charged amino acids were omitted or kept at very low frequencies for CDR2 and CDR3 respectively, as a high frequency of these have been linked to promotion of poly-reactivity (lack of specificity) and increased nucleic acid binding of the antibodies [16,40]. The CDR2 was designed to have a low diversity by limited randomization of only four positions (Figure 3). This entails that the overall diversity is minimized, thus ensuring that the total library diversity can be sampled in the phage display system. The choice of amino acids and their frequencies in the randomized positions were based on refolding studies of dAbs [6]. Due to the genetic rearrangements underlying the natural immune repertoire it is the CDR3 loop in the antibody repertoire which displays the highest diversity. Therefore the CDR3 loop in Predator library was designed to take this into account by randomizing seven positions. The amino acid compositions of the randomized positions of the CDR3 were based on an analysis of the amino acid frequencies in the heavy chain CDR3 of functional human antibodies [41]. Alanine residues were omitted in most positions in CDR3, as it has been suggested that their main function is to convey flexibility to the loop, and that serine and especially glycine are more suitable for this [40]. Isoleucine, methionine and lysine were not used in CDR3 as these amino acids are generally rare in the functional CDR3 [41]. They furthermore possess features we were looking to minimize i.e. positive charge and hydrophobicity. As the use of the phenylalanine, aspartic acid and tyrosine (FDY) motif is so prominent at the end of the heavy chain CDR3 in functional human antibodies, these three amino acids were used instead of the amino acids found in the positions in the HEL4 antibody [41]. Finally cysteine residues were excluded from the CDR3, as the formation of disulfide bridges by free cysteine residues in oxidizing environment would likely cause problems (Figure 3). In nature the length of the CDR regions, especially CDR3, have been correlated to the type of antigen bound. Antibodies binding large protein antigens often have a short CDR3, thus creating a flat binding surface while antibodies binding small antigens and haptens often have longer CDR3 regions [42]. Yet, a longer than average CDR3 is more prone to structural instability and polyreactivity, if not stabilized [41,43]. As a long CDR3 is not a feature of the HEL4 antibody, the Predator library employs a medium sized CDR3 of 10 amino acids (Kabat numbering). To ensure that the library would encompass the most desirable combinations of amino acids with regard to the theoretical binding characteristics we assigned frequencies according to the above cited literature. This means that highly hydrophobic or highly positively charged CDR3s might be present, but they should constitute only a small portion of the library.

### Library assembly

**Figure 4:**
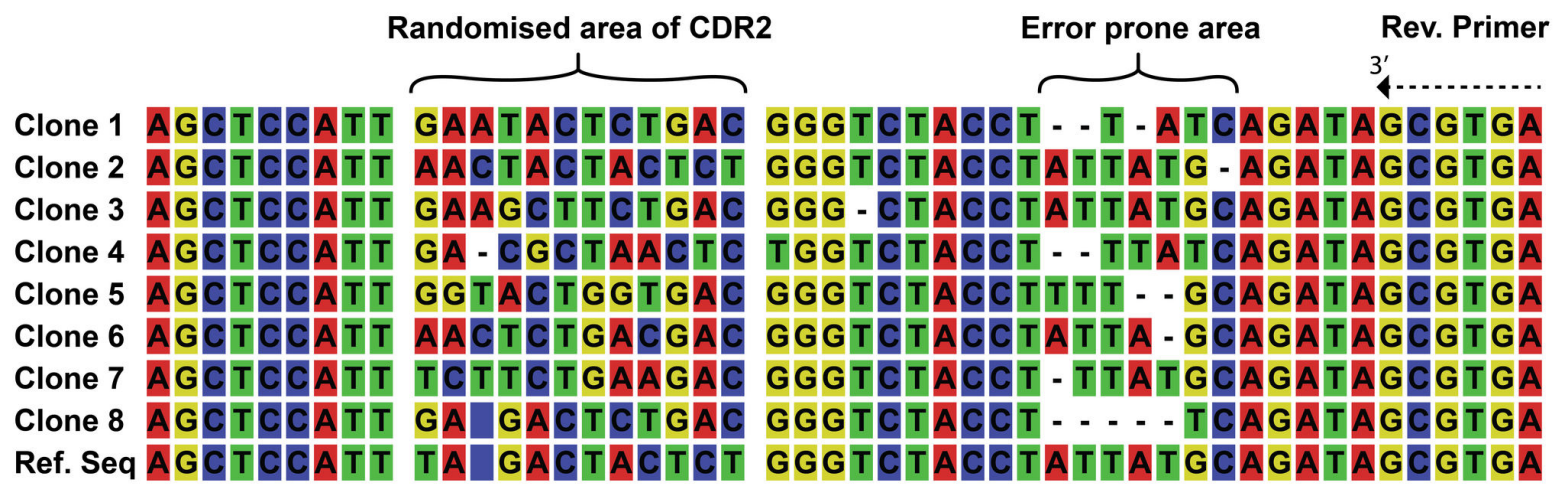
Sequences showing an error prone repeat close to primer annealing site. The sequencing data from eight clones with errors and a reference sequence showing the CDR2 and immediate downstream region. The nucleotides composing the randomized region of CDR2 are indicated at the top along with the nucleotides matching the 3’ end of the reverse primer used for the generation of the CDR2 insert. Eight of the shown clones have errors and all of the errors have occurred in the area between the randomized region of CDR2 and 3’ end of the reverse primer used in the generation of the CDR2 insert. Especially the “TATTAT” repeat seems susceptible to errors.

**Figure 5:**
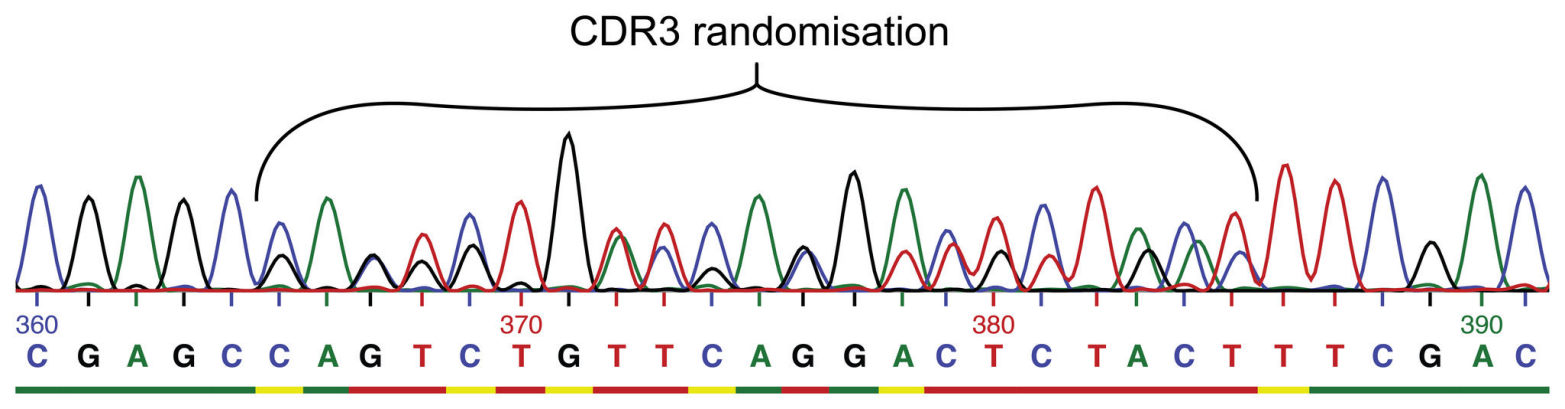
Trace data from sequencing of “double read” clone. The trace data from the CDR3 randomized region of one of the clones we define as having a double read. We found the trace data to be unusual as there was a perfect read both before and after the CDR3 region. Furthermore the trace data indicate that there are two very clearly defined reads mixed together in the region.

The CDR regions were synthesized as single stranded DNA oligonucleotides by trinucleotide synthesis, and a cycle free polymerase second strand synthesis was performed. That the extensions were made without cycling of the temperature should reduce the risk of clonal bias, e.g. from random or sequence related differential amplification efficiencies. The library was generated in two steps with CDR2 being inserted into the vector first. After amplification and purification of the vector containing diversity in CDR2, the CDR3 were inserted by restriction digestion and ligation. The CDR2 sub-library had a diversity of more than 10^5^. Based on the frequencies of amino acids used this should theoretically cover the rarest clones in the CDR2 more than two times. After insertion of the CDR3 region in the CDR2 sub-library, the Predator library was estimated to have a diversity of more than 7.8 x 10^7^ different clones. Some of the amino acids in CDR2 and CDR3 are presented at very low frequency. However if we only consider the most common combinations found in CDR2, these should statistically be present one time for every 334 clones and the most common combination found in the CDR3 should be present one time for every 727,000 clones. This means that the most common combination of CDR2 and CDR3 will statistically be present at least one time for every 2.43 x 10^8^. After the library construction, 24 clones were isolated and sequenced. Of these 24 clones, 14 were verified as good sequences, two failed in sequencing, and eight had various frameshifts, presumably due to errors in the synthesis of dsDNA. The errors were clustered near the reverse primer annealing site downstream of CDR2 (Figure 4). None were found in the CDR3 area. None of the failed clones were capable of producing fusion pIII when investigated on the DNA level. Among the 14 clones verified to harbor correct plasmid, five of the clones gave “double” reads in sequencing for the CDR3 area, but never in the CDR2 (Figure 5). This was speculated to be a result of dissociation and non-homologous annealing in the synthesis method used for the CDR3 inserts. We used the isolated plasmid from one of these clones to transform cells and a re-sequencing supported our hypothesis. The colonies fell into two groups, each one having a sequence matching the trace data from the first sequencing. None of the “second generation” clones showed any errors at the DNA level. If we draw the conclusion that the double reads in the sequencing trace data are due to a single transformation event with a parent plasmid, in which the CDR3 insert had non-homologously annealed during the second strand synthesis, then the progeny of this transformant will eventually harbor only one of the two plasmids. Including this assumption in the calculation of the library size we arrive at a functional library size of 6.2 x 10^7^.

To see how any errors might influence on a selection the library was packaged using KM13 helper phages and treated with trypsin before infection into log-phase TG1. Ten colonies from the infection were picked and sequenced and all sequences were unique and without errors indicating that the errors seen are not likely to affect the functionality of the library (data not shown).

Phage particles were produced from the library using KM13 and dilution series for titration were made to test the efficiency of phage production. The yield of phage particles reached 10^12^ to 10^13^ after two rounds of PEG precipitation which is in line with results from other phagemid libraries (data not shown). In parallel with the titration of the library, the dilution series was digested with trypsin to remove KM13 derived pIII from phage particles, and thereby rendering non-displaying phage particles un-infective. The display level was estimated to be 6.4% based on the number of colonies appearing after infection with trypsin treated and untreated phage. Smaller proteins are generally better displayed on the phage proteins and likely to express better as soluble protein. That the library is a synthetic library, as opposed to a naïve or immunized library, may show to have both benefits and drawbacks. Clones selected may show to be less functional as they have not been through a natural *in vivo* selection for functionality. On the other hand the use of a single well tested framework should hopefully reduce such problems; furthermore the use of a single framework may help to reduce some of the growth differences between the clones [44]. This is corroborated by the use of only one codon for each amino acid in the CDR randomizations, which also reduces the differences between the clones at the DNA level. Furthermore there is no amber stop codons present in the library which is a problem often negatively impacting the functionality of libraries produced using for example NNK randomization. Due to the known frequencies of all individual amino acids in the CDRs, and the amplification free second strand synthesis, we can be confident that the functional diversity of the Predator library of around 6.2 x 10^7^ is by and large comprised of unique clones. Although the overall library size is an important factor for obtaining high affinity clones, issues like the functional size of the libraries and the quality of the clones selected from the libraries have recently attracted increasing focus [45].

### Selections on purified protein

**Figure 6:**
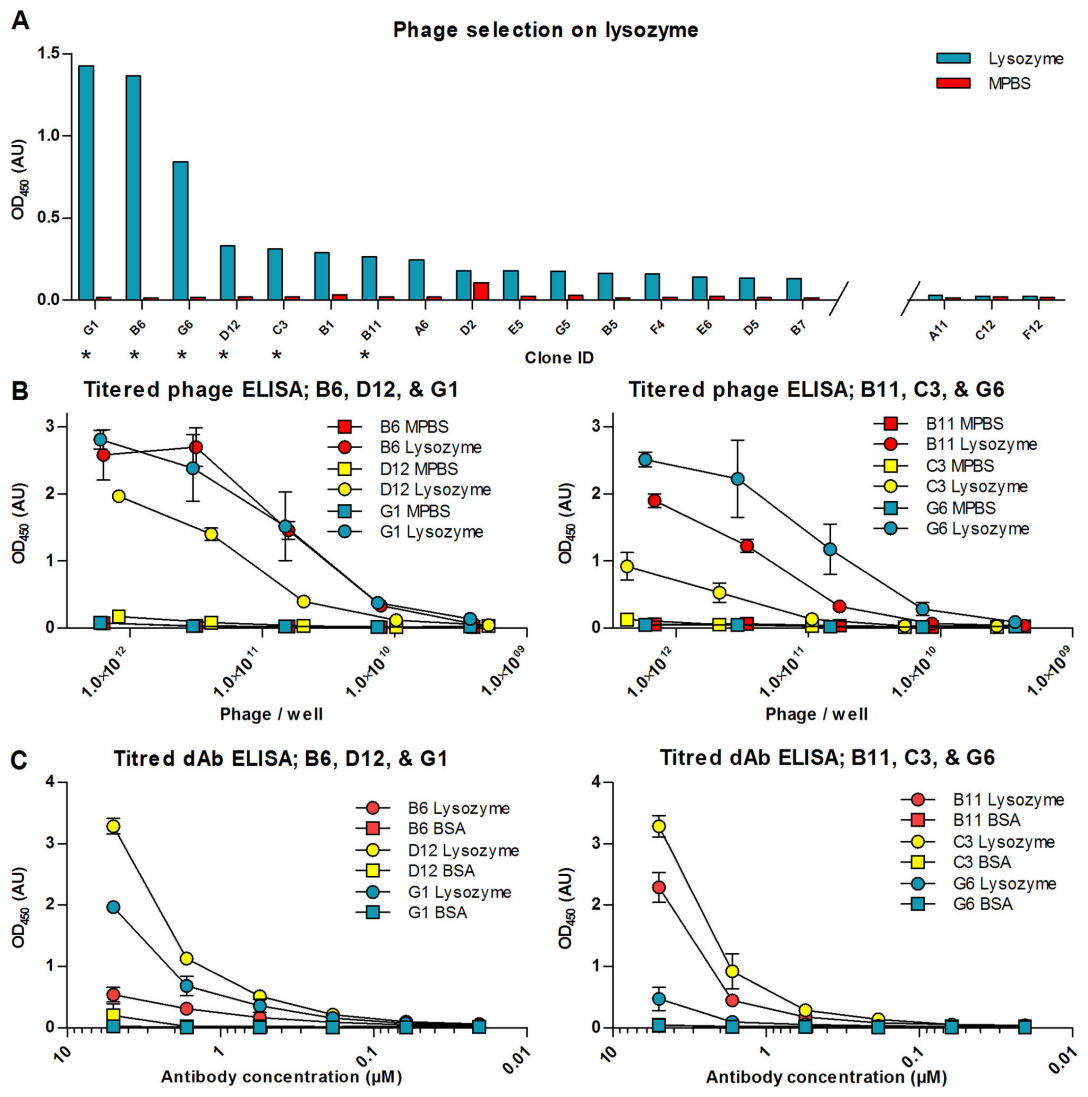
Screening ELISA from selection on lysozyme and antibody-phage ELISA dilution series on lysozyme. (**A**) The clones with the highest signal on immobilized lysozyme are shown in the figure. The signal on lysozyme is depicted as blue bars, whereas the signal on milkprotein is shown with red bars. The three clones with the lowest signal among the 96 clones screened are also shown for comparison. Clones marked with an asterisk were examined further in titered phage ELISA, ELISA using soluble domain antibodies and in western blots. (**B**) Six of the clones from the screening ELISA were tested further in a titered phage ELISA in microwells. The y-axis of the graphs depicts the signal for serial dilutions of the phages displaying the selected domain antibodies, and the x-axis depicts the phage titter in the wells of the dilution series. The binding to milk proteins is indicated with boxes, while the binding to lysozyme is indicated with circles. The error bars show one standard deviation based on three independent experiments. (**C**) The six clones were also tested in an ELISA using soluble antibody fragments. Here the x-axis depicts the concentration of antibody added to respective wells of the dilution series. The error bars show one standard deviation based on three independent experiments.

In order to verify the functionality of the library, a selection on purified protein was carried out. After a single round of panning on lysozyme 95 clones were isolated. When screened in monoclonal antibody-phage ELISA to identify potential lysozyme binding clones, a number of clones were noted to give good signal on lysozyme and not on milk protein which was used for blocking free plastic surfaces (Figure 6A). Based on the ratio of signal on lysozyme over the signal on milk protein, the clones B6, B11, C3, D12, G1, and G6 were analyzed further. Initially a titration series ELISA was performed using antibody-phage. In this ELISA the recognition of lysozyme was compared to the signal on milk protein for varying antibody-phage dilutions. The antibody genes were sub-cloned into pET22b and expressed without fusion to protein III before testing in titration ELISA against lysozyme. The experiments confirmed that all six clones were indeed binding specifically to lysozyme (Figure 6B & C). Furthermore, western blots using antibody-phage were carried out, and all six clones gave rise to a single band at 14 kDa, which is consistent with the recognition of lysozyme. The band was sharpest for the clones D12 and G6 (data not shown), but could be identified for all six clones. Finally, all the six clones were sequenced, proving that all of the six selected clones are in fact unique (Data not shown).

### Characterization of selected clones

**Figure 7:**
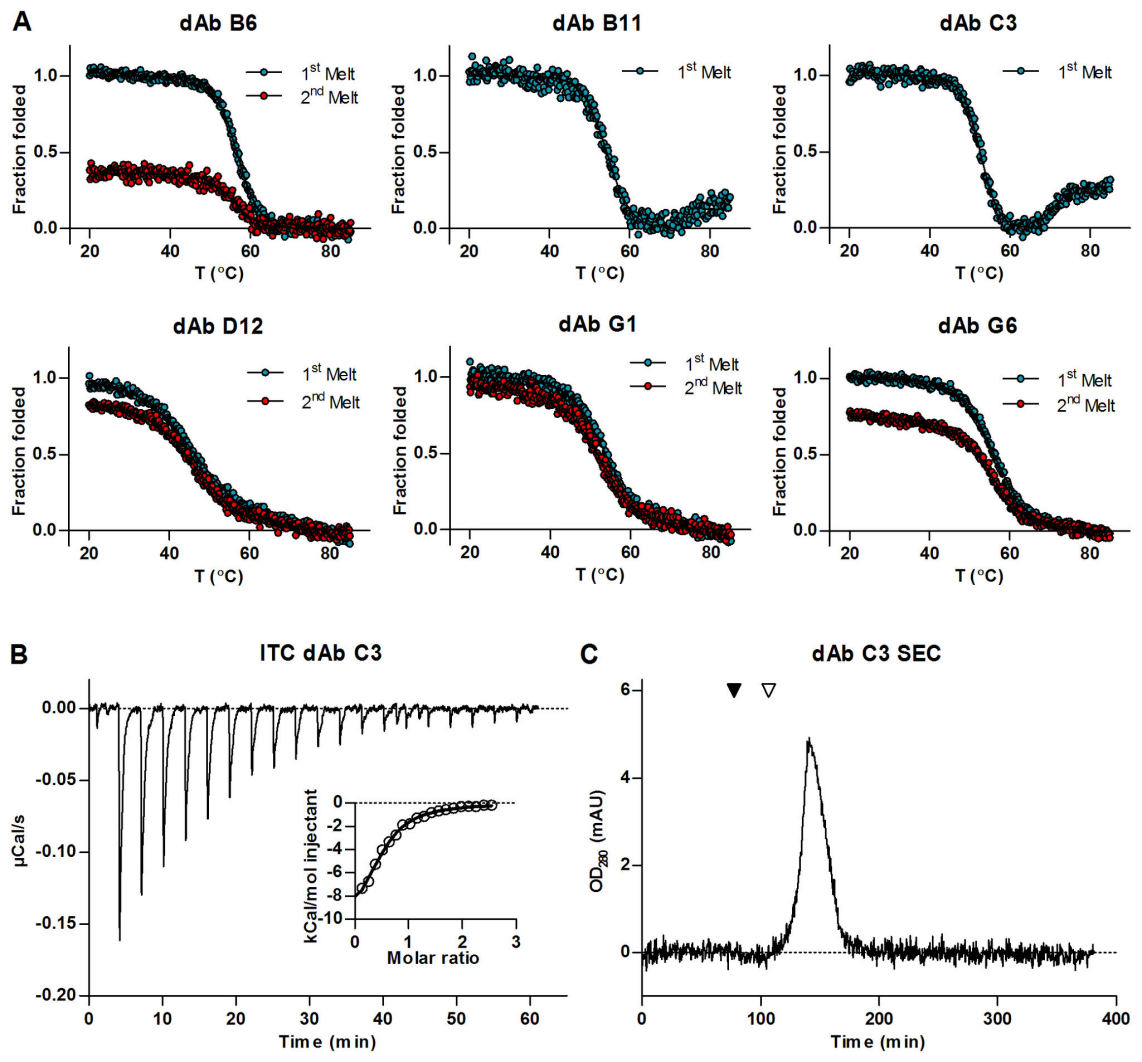
Biophysical evaluation of isolated antibodies and affinity measurement for C3 against hen egg lysozyme. (**A**) Thermal unfolding and refolding for the lysozyme binding clones assayed by circular dichroism spectroscopy. The first melting curve for each clone is shown in blue, and the subsequent melting curve in red. After recording the first melting curve the antibodies were allowed to cool, and the experiment was repeated using the refolded antibodies. (**B**) Recorded heat change over time during successive injections of lysozyme into the cell containing C3 domain antibody. The insert shows the integrated injection heats as a function of molar ratio of the lysozyme to the antibody. Circles are the integrated signal from the larger panel, and the best fit of the 1:1 binding equation to the observation is shown as a line. The figure is representative for the three measurements performed. (**C**) Chroatogram from size exclusion chromatography of C3 antibody. The migration of two size markers is shown in the chromatogram; albumin (black triangle) with a size of 66 kDa, and TEV-protease (white triangle) with a size of 26 kDa. The single elution peak of the antibody well below the 26 kDa reference protein indicates that it exhibits a monomeric state in solution.

For further testing, the six clones were expressed in 1 liter cultures. The expression level ranged from 4 mg/l to 15 mg/l for the six clones tested. The antibodies were purified using protein A columns and dialysed into PBS. The thermodynamic stability of all six clones was measured using CD spectrometry (Figure 7A). Melting temperatures for all six clones fell within 52°C to 56°C, except the clone D12, which had a melting temperature of 45.9°C. After heating to 85°C and cooling to 25°C, four of the six clones appeared to refold to the original secondary structure to varying degrees and two of the clones appeared to be irreversibly unfoldeded or aggregated. In the case of G1 and D12, the first and second melting curves overlap, suggesting that the protein is able to fold back to its original conformation after thermal unfolding. In the cases of B6 and G6, the curves indicate varying degrees of refolding, but not a complete return to the nascent structure. The antibodies B6, D12, G1, and G6 did not aggregate upon heating, whereas B11 and C3 aggregated irreversibly, precluding recording of a second melting curve. There were no systematic differences in either the pI or the occurrence of hydrophobic residues in CDRs between the clones. The results show that the addition of the I29D mutation to the HEL4 scaffold strongly reduces the thermodynamic stability of the dAbs. The introduction of a D at position 29 have however not been shown to reduce the melting temperature compared to HEL4 [11]. Both position 29 and 31 are part of the upper core residues in the V_H_ fragment and are normally occupied by bulky hydrophobic amino acids [22]. It is likely that the reduction of thermodynamic stability seen when combining the I29D mutation with “D” at position 31 of the HEL4 scaffold is a result of the simultaneous changing of two central upper core residues. It has previously been reported that the introduction of three hydrophilic mutations at positions 44, 45 and 47 increased solubility but decreased the thermodynamic stability [9]. The results further show that the composition of CDR2 and CDR3 can have an decisive impact on the refolding and aggregation propensity which may be more pronounced than previously reported [6,11]. We further investigated the binding characteristics in solution of the clone C3 to lysozyme by the use of isothermal titration calorimetry (ITC). An affinity constant (K_D_) of ~5 µM (Figure 7B) was calculated by fitting a 1:1 binding model to the binding curves. The ITC measurements furthermore indicated that the antibody was 77% active. In size exclusion chromatography the C3 domain antibody elutes as a single sharp peak, thus giving no indications that dimers or higher order aggregates are forming (Figure 7C). It is, however, known that commercial available lysozyme may contain dimers which could influence binding curves [46].

### Selection on whole cells

**Figure 8:**
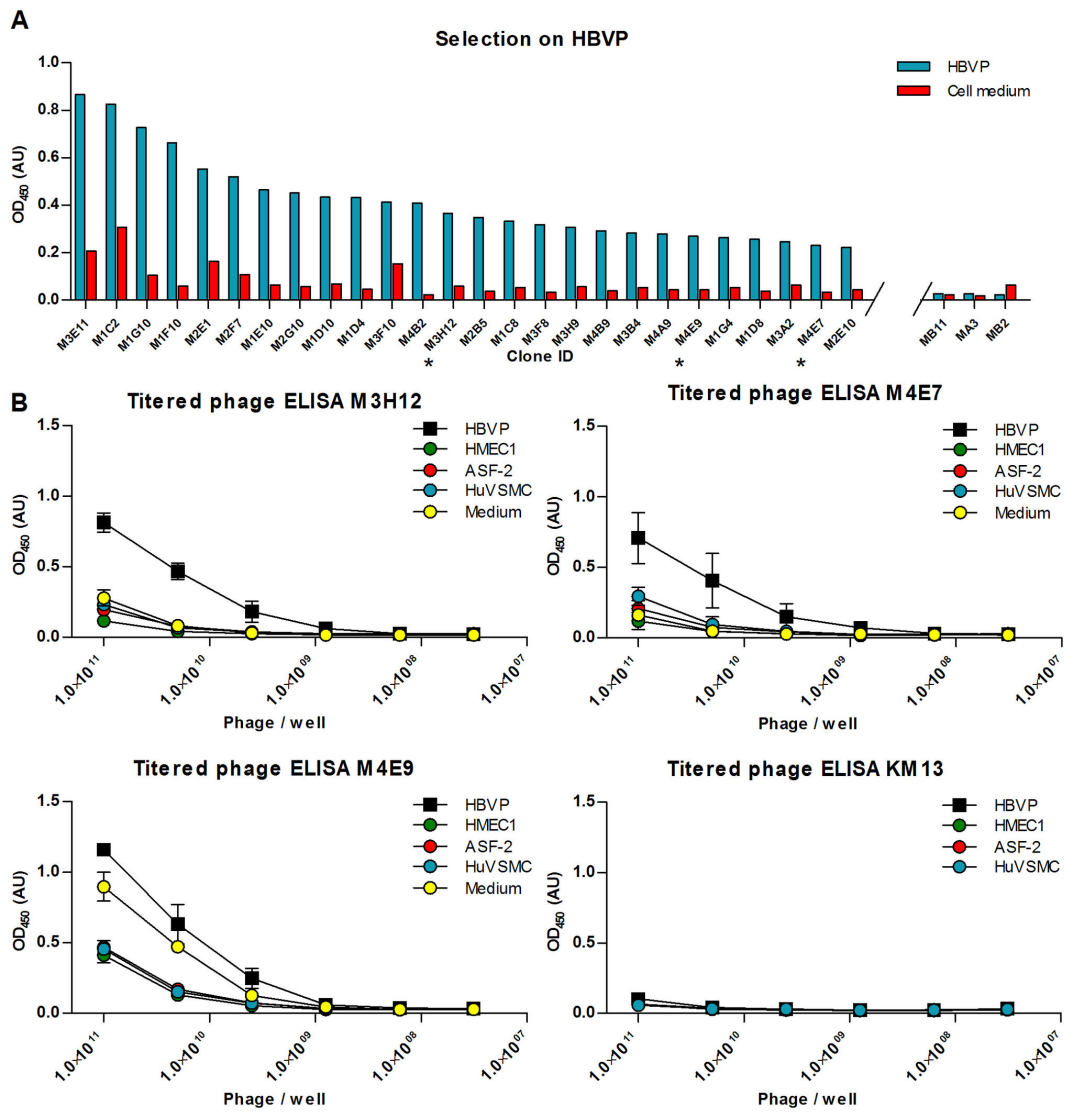
Screening ELISA from selection on HBVP cells and antibody-phage ELISA dilution series on HBVP. (**A**) The clones shown are the ones for which the signal on HBVP-cells was highest, and for which the signal on cell medium did not exceed 25% of the signal on cells. The three clones with the lowest signal among the 384 clones screened are shown for comparison. Blue bars depict the signal on HBVP cells, whereas signal on medium is depicted in red. The result was obtained after one round of selection on immobilized HBVP cells. Clones marked with an asterisk were examined further in titered ELISA. (**B**) Three of the clones from the screening ELISA were tested further in a titered phage ELISA in microwells. The x-axis of the graphs depicts the titer for serial dilutions of the phages displaying the selected domain antibodies, and the y-axis depicts the ELISA signal. The signal on HBVP cells is marked with boxes, while the signal on various decoy cells and on cell medium is marked with circles. The error bars show one standard deviation calculated on the basis of three independent experiments.

The use of the smaller dAb format could turn out to be an advantage for the selection on whole cells, as the smaller antibody fragment may penetrate better in between the many molecules on the surface of the cell [47]. We therefore challenged the Predator library in selections on intact HBVP cells. A single round of panning was performed and 384 clones were picked and screened in ELISA for binding to HBVP (Figure 8A). Of the 384 clones, three clones were tested further in dilution series antibody-phage ELISA for their specificity for HBVP compared to other relevant cell types. The dilution series ELISA results show a clear preference of two of the clones for HBVP cells over other cell types, while the third clone specifically recognized a component of the HBVP medium not found in the media used for the other cell types (Figure 8B).

**Figure 9:**
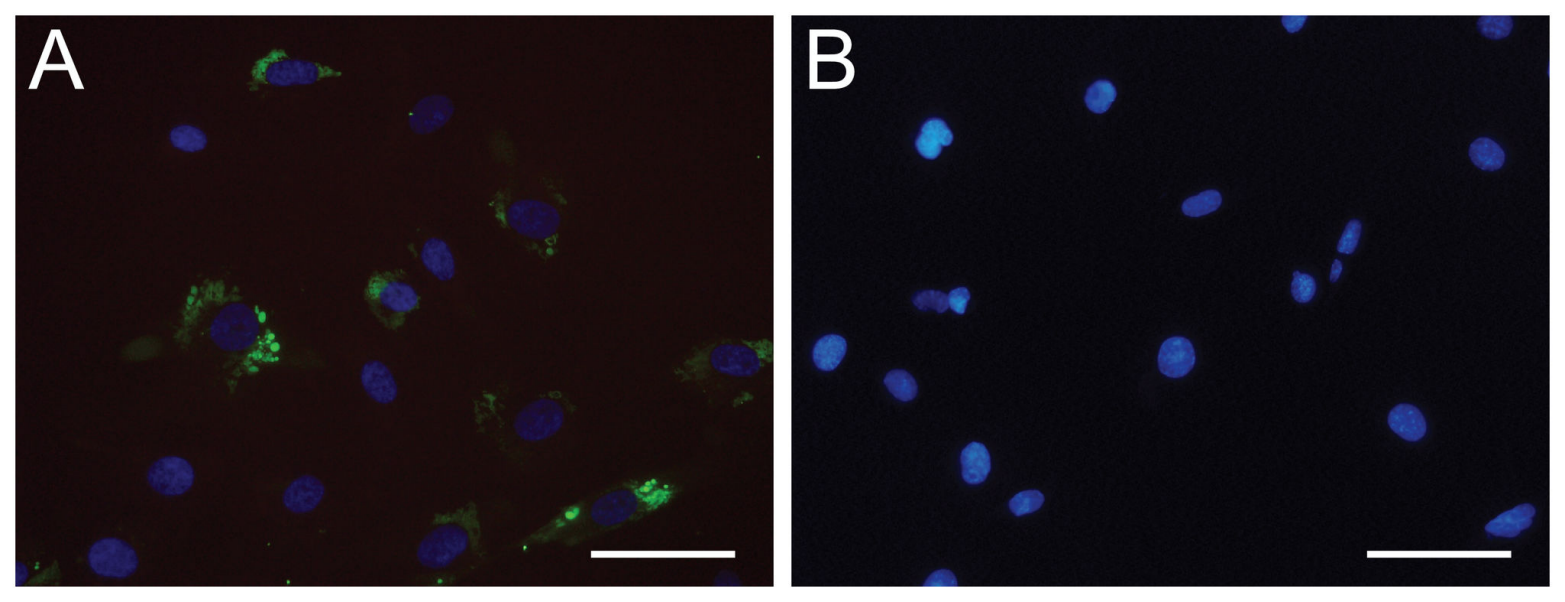
Cell staining with soluble M3-H12 Predator antibody. The cell lines HVBP (A) and HMEC-1 (B) were stained with selected M3-H12 antibody and detected with Protein A conjugated with Alexa Fluor 488 (green). The cell nuclei were stained with DAPI (blue) (scale bar, 20 µm).

The clones recognizing HBVP cells specifically were sub-cloned and expressed without fusion protein. The purified antibodies were used for immunocytochemistry on cells and the staining confirmed the specificity of the selected antibody for HBVP cells. The staining furthermore revealed that the selected clones seemed to recognize a sub-population within the HBVP cell line (Figure 9). Western blots were made using HBPV cell lysate and the purified antibodies, but no bands were detected. This could be due to too low amount of antigen in the sample or the disruption of conformational sensitive epitopes upon cell extraction (data not shown).

## Conclusions

In this paper we describe the creation of a novel phagemid domain antibody library dubbed Predator with a diversity of approximately 6.2 x 10^7^ unique functional clones. For the construction of the Predator library we have been building on some of the established ideas already used in existing libraries like the modular built and the use of trinucleotides for the CDR randomizations [18,26]. As a novel feature the library diversity was synthesized and elongated by a cycle free polymerase reaction. Also the aggregation resistant domain antibody framework has been used for libraries [28]. The new combination of the established ideas and the novel design of the CDR randomizations including the specific frequencies assigned to the different amino acids at each position, however, make the Predator library stand out from other libraries presently available. Sequencing of phage derived clones packaged using KM13 and treated with trypsin to simulate a selection elution further showed that frameshifted clones encountered in the primary titering will be rendered non-infective when trypsin elution is used in combination with KM13 rescue of the library. The library was successfully used for single round selection on both purified protein and whole cells yielding several specific binders. However, further analysis of some of the selected clones showed that the introduction of a hydrophilic “D” residue at position 29 of the HEL4 scaffold caused a reduction in the thermodynamic stability of the antibody fragments.

Regarding the selection on purified protein we show that the antibody library is functional, and that using a single round of stringent selection in combination with KM13 helper phage several unique binders can be selected. The affinity of a selected clone was determined to be 5µM. However, it should be noted that only one round of selection was applied, therefore the enrichment for high affinity binders were low and only a limited number of the resulting phage antibodies were chosen for further analysis. Furthermore, the modular design of the library affinity maturation could easily be made by e.g. CDR walking. The results that we see from the selection on lysozyme are in agreement with other selections that have been performed using the library. Specifically, the library has been used in selections on a purified serine protease of human origin, on a purified recombinant histone deacetylase from trypanosomatid protozoa, and on various native and recombinant human complement proteins.
